# Multidisciplinary management of refractory Kawasaki disease with bilateral giant coronary artery aneurysms and latent tuberculosis infection: a case report focusing on immune–coagulation–infection crosstalk

**DOI:** 10.3389/fimmu.2026.1796288

**Published:** 2026-03-11

**Authors:** Bingqian Xue, Mengjun Luo, Yang Wen, Yanfeng Yang, Hua Lai, Yizhou Wen, Qin Zhou

**Affiliations:** 1 Department of Clinical Medical Laboratory, The Third People’s Hospital of Chengdu, Chengdu, Sichuan, China; 2 Department of Clinical Laboratory, Chengdu Women’s and Children’s Central Hospital, School of Medicine, University of Electronic Science and Technology of China, Chengdu, Sichuan, China; 3 Department of Ultrasonography, Chengdu Women’s and Children’s Central Hospital, School of Medicine, University of Electronic Science and Technology of China, Chengdu, Sichuan, China; 4 Department of Pediatric Cardiology, Chengdu Women’s and Children’s Central Hospital, School of Medicine, University of Electronic Science and Technology of China, Chengdu, Sichuan, China; 5 Department of Radiology, Chengdu Women’s and Children’s Central Hospital, School of Medicine, University of Electronic Science and Technology of China, Chengdu, Sichuan, China

**Keywords:** refractory KD, giant CAAs, LTBI, multidisciplinary management, precision anticoagulation

## Abstract

**Background:**

The management of refractory Kawasaki disease (KD) with giant coronary artery aneurysms (CAAs) complicated by latent tuberculosis infection (LTBI) represents a formidable “triple challenge”: it requires balancing aggressive inflammation control against the risk of tuberculosis reactivation, managing the extreme cardiovascular risk of giant CAAs driven by immune-coagulation crosstalk, and navigating the pharmacological hurdles of LTBI prophylaxis, namely rifampicin-induced warfarin resistance. Developing a safe, integrated strategy to concurrently address these inflammatory, cardiovascular, and infectious risks remains an urgent and unmet clinical need.

**Case presentation:**

We report a 3.5-year-old boy with refractory KD and rapid-onset bilateral giant CAAs. Pre-biologic screening identified LTBI, strictly contraindicating guideline-recommended tumor necrosis factor-alpha (TNF-α) inhibitors. A multidisciplinary team (MDT) executed a sequential, pathophysiology-driven protocol: 1) Immunomodulation & Prophylaxis: Systemic inflammation was controlled using methylprednisolone and a second IVIG dose, circumventing TNF-α blockade to ensure LTBI safety. Upon vasculitis remission and hepatic recovery, LTBI prophylaxis (isoniazid/rifampicin) was initiated. 2) Precision Anticoagulation: To overcome rifampicin-accelerated warfarin metabolism, we executed a rapid, percentage-based titration alongside a “prolonged low-molecular-weight heparin (LMWH) bridge”. A stable, conservative therapeutic INR (1.5–2.5) was achieved, requiring an exact 2.5-fold warfarin dose escalation. At the 9-month follow-up, the CAAs demonstrated luminal normalization, though necessitating lifelong surveillance for vascular pseudonormalization.

**Conclusion:**

This case illustrates how an MDT-led, pharmacology-guided approach effectively resolves therapeutic conflicts in high-risk KD with biologic contraindications. By integrating alternative immunomodulation, strategic prophylaxis, and precision anticoagulation, this framework provides a practical clinical paradigm for managing intricate immune-coagulation-infection crosstalk.

## Introduction

Kawasaki disease (KD) is an immune-mediated acute systemic vasculitis of unknown etiology, mainly affecting children under 5 years old and is the leading cause of acquired heart disease in children in developed countries (1, 2). Timely treatment with intravenous immunoglobulin (IVIG) and high-dose aspirin achieves clinical remission in 80–90% of patients and reduces the incidence of CAAs from 18% to 4% (2, 3). However, approximately 10–20% of children develop refractory KD, characterized by persistent or recurrent fever after initial IVIG therapy, and these individuals face a markedly elevated risk of CAAs (4). As a rare but severe sequela of KD, giant CAAs (*Z* score ≥10 or absolute dimension >8 mm) expose patients to lifelong risks of thrombosis, stenosis, myocardial infarction, and sudden cardiac death, driving substantial long-term morbidity and mortality (2). Despite the clinical indication for intensified therapy in such severe cases, the selection of immunomodulators [e.g., glucocorticoids (GC), tumor necrosis factor-alpha (TNF-α) inhibitors, cyclosporine] remains dependent on clinician judgment due to the lack of head-to-head comparative trials (1, 2), and there are no standardized clinical guidelines for this population.

The management of refractory KD is further complicated by latent tuberculosis infection (LTBI), which poses a significant therapeutic dilemma. As a significant global health issue, particularly among exposed pediatric populations, LTBI, which refers to a condition in which the body mounts a persistent immune response to *Mycobacterium tuberculosis* antigens in the absence of clinical or radiological evidence of active tuberculosis (5), presents a clinical challenge when it contraindicates the use of biologic agents (6). Although TNF-α inhibitors like infliximab are recommended second-line therapies (1), they are strictly contraindicated in LTBI-positive patients due to the risk of granuloma disintegration and tuberculosis reactivation (7). This necessitates alternative immunomodulatory strategies; however, evidence regarding the efficacy of such alternatives in patients with concurrent giant CAAs and LTBI is extremely scarce.

Furthermore, giant CAAs require long-term anticoagulation, typically with warfarin (1). When combined with LTBI prophylaxis using rifampicin, a potent cytochrome P450 inducer, significant pharmacokinetic interactions occur. Rifampicin accelerates warfarin metabolism, leading to “warfarin resistance” and making therapeutic International Normalized Ratio (INR) maintenance exceptionally difficult (8, 9). Thus, frequent and substantial dose adjustments of warfarin are often required to maintain a therapeutic INR (10).

Herein, we report a 3.5-year-old boy with refractory KD, bilateral giant CAAs, and LTBI, who faced a “triple challenge” including immunomodulatory restrictions, high thrombotic risk, and rifampicin-warfarin interaction. We detail a multidisciplinary, sequential therapeutic strategy consisting of rescue IVIG, GC, and pharmacokinetic-guided warfarin titration that successfully achieved inflammation control, aneurysm stabilization, and infection safety. This case provides a practical paradigm for managing complex immune-coagulation-infection crosstalk in high-risk KD.

## Case description

**Figure 1:**
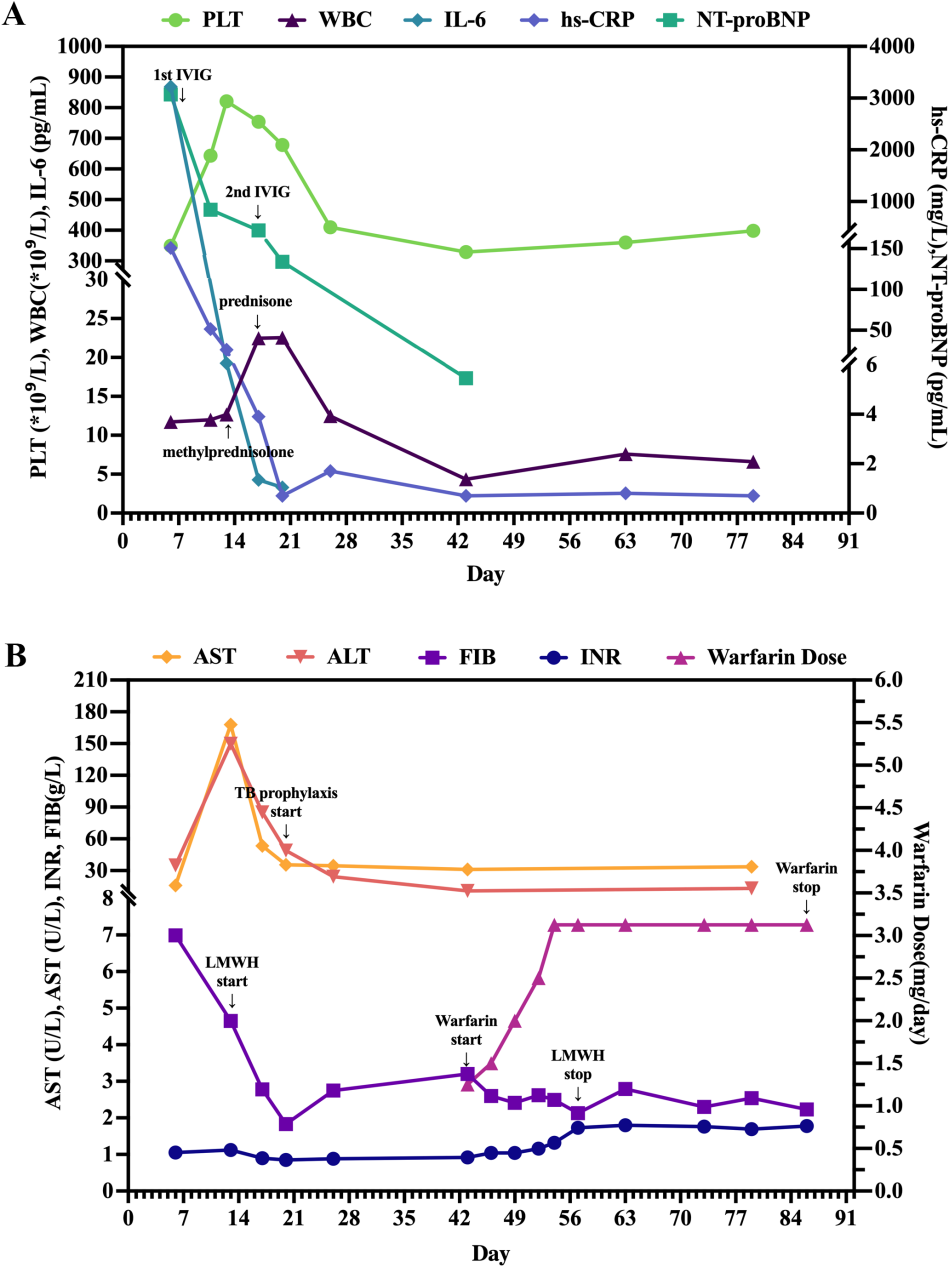
Serial laboratory biomarkers and anticoagulation management. **(A)** Temporal changes in inflammatory and cardiac biomarkers, including platelet count (PLT), white blood cell count (WBC), interleukin-6 (IL-6), high-sensitivity C-reactive protein (hs-CRP), and N-terminal pro-B-type natriuretic peptide (NT-proBNP). **(B)** Trends in liver function indices (aspartate aminotransferase [AST] and alanine aminotransferase [ALT]), coagulation parameters (fibrinogen [FIB] and international normalized ratio [INR]), and warfarin dosage titration. The timeline illustrates the pharmacology-informed dose adjustments required to overcome rifampicin-induced warfarin resistance, alongside the resolution of transient hepatotoxicity.

**Figure 2:**
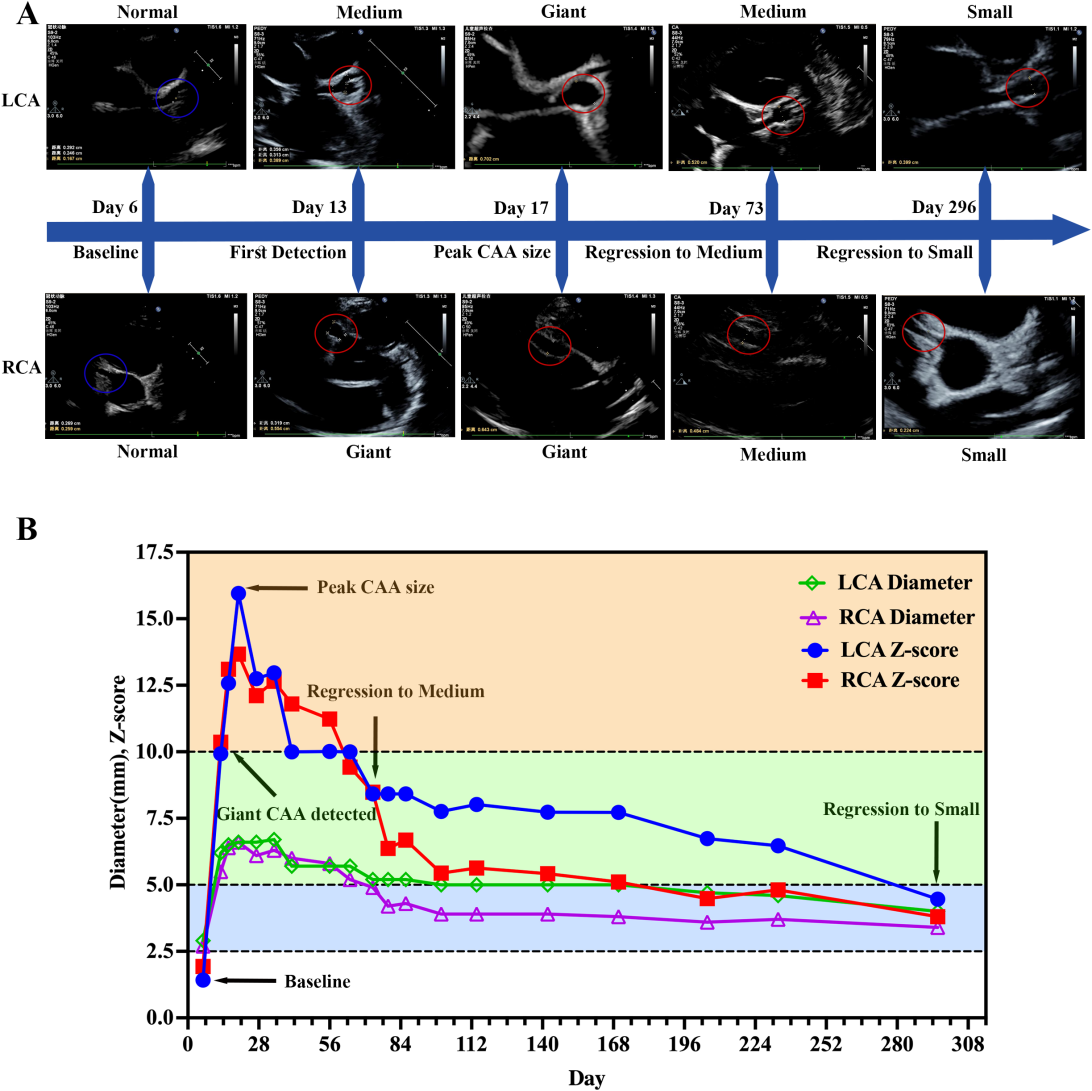
Assessment of coronary artery aneurysms (CAAs) progression and regression. **(A)** Serial echocardiography images of the left coronary artery (LCA) and right coronary artery (RCA) at critical milestones: baseline (Day 6), initial diagnosis of giant CAA (Day 13, Z score ≥ 10), peak dilation (Day 16), and progressive regression (Days 73 and 296). Baseline normal segments are indicated by blue circles; dilated segments are highlighted by red circles. **(B)** Dynamic trends in LCA/RCA diameters and Z-scores, highlighting the transition from giant to small CAA stages.

A previously healthy 3.5-year-old boy (15 kg) was admitted on March 2, 2025 (Day 5) for 4 days of persistent fever (peak 40 °C, poor response to antipyretics) and classic KD manifestations, including non-purulent conjunctival injection, strawberry tongue, and cervical lymphadenopathy. Admission laboratory findings confirmed a systemic hyperinflammatory and hypercoagulable state with hepatic involvement (**Figure 1**). A baseline echocardiogram (ECHO) performed on Day 6 showed no coronary artery dilation (**Figure 2**). Following initial treatment with IVIG (2 g/kg) and high-dose aspirin on Day 7, symptoms temporarily resolved, leading to a brief discharge on Day 12. However, follow-up ECHO on Day 13 revealed rapid coronary progression; the right coronary artery (RCA) reached giant aneurysm dimensions (Z = 10.36), while the left coronary artery (LCA) showed a medium-to-large aneurysm (Z = 9.93). The longitudinal trajectory of coronary Z-scores and diameters demonstrated a peak on Day 20, followed by progressive regression (**Figure 2**). Serial laboratory monitoring was continuously performed throughout the acute and follow-up phases (**Figure 1**).

**Figure 3:**
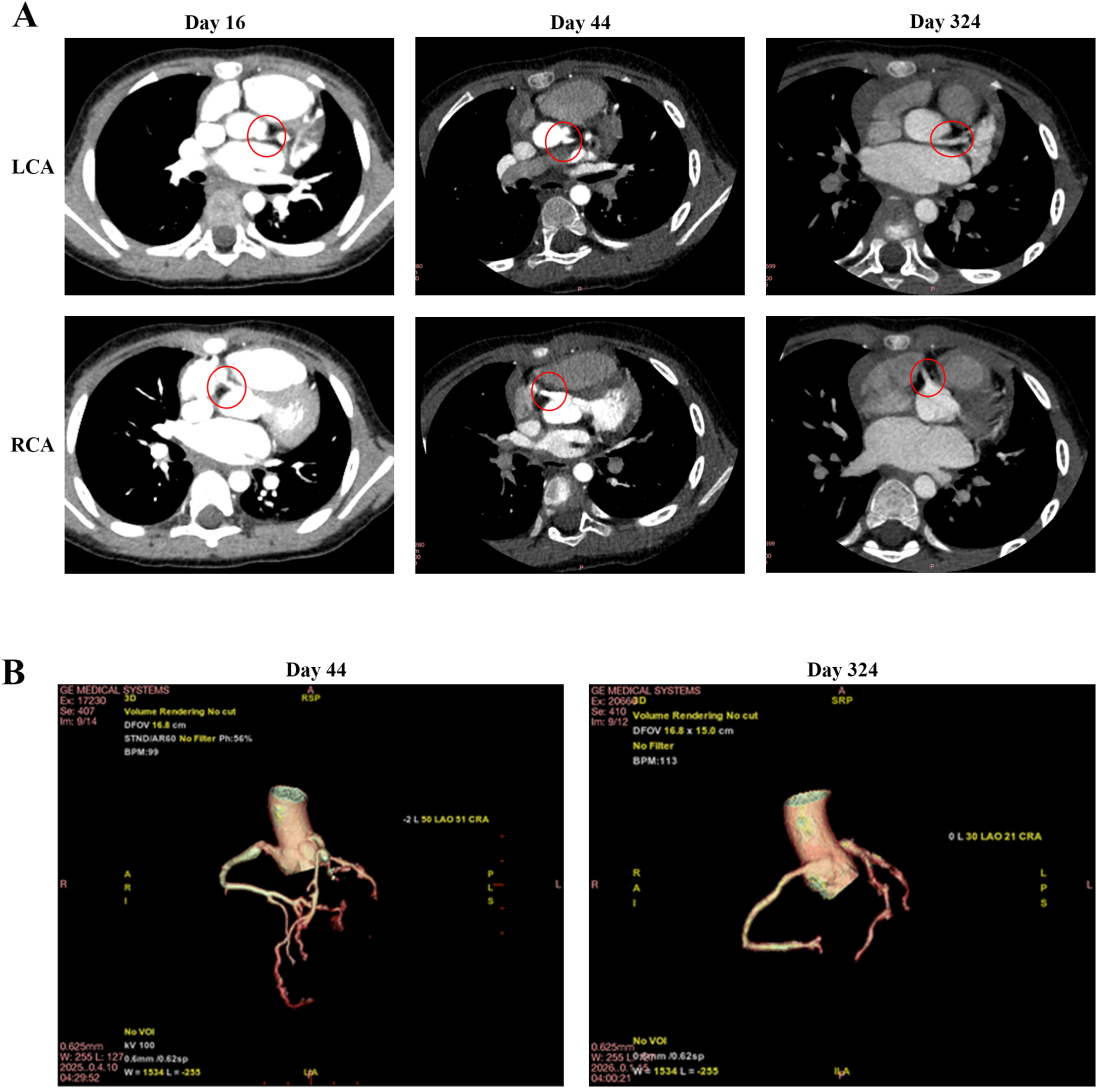
Radiological evaluation of giant CAA regression via coronary computed tomography (CT). **(A)** 2D axial imaging: Baseline plain CT (Day 16) and subsequent coronary computed tomography angiography (CCTA) (Day 44) demonstrate bilateral giant aneurysms (red circles). Follow-up imaging at Day 324 confirms complete morphological normalization with smooth vascular contours. **(B)** 3D coronary reconstruction: Spatial morphology of the coronary tree at Day 44 visualizes the extent of bilateral giant aneurysms. Follow-up reconstruction at Day 324 demonstrates anatomical restoration without residual stenosis or aneurysmal dilation.

Prior to initiating second-line biologics, safety screening (Day 13) unexpectedly yielded a positive QuantiFERON-TB Gold Plus (QFT-Plus) result. A plain chest computed tomography (CT) scan was obtained on Day 16, which revealed bilateral CAAs with no evidence of active pulmonary tuberculosis (**Figure 3A**). Lacking a known tuberculosis exposure history, the patient was diagnosed with LTBI, thus contraindicating the use of TNF-α inhibitors. An urgent multidisciplinary team (MDT) comprising pediatric cardiology, rheumatology and immunology, gastroenterology, infectious diseases and clinical pharmacy specialists was convened, and a sequential intensive immunomodulatory regimen was formulated. The patient received intravenous methylprednisolone (15 mg q12h, starting Day 13) and a second IVIG dose (2 g/kg, Day 17), which rapidly normalized inflammatory and cardiac function biomarkers (**Figure 1**). Upon vasculitis control and hepatic recovery (Day 20), LTBI prophylaxis (isoniazid plus rifampicin) was initiated. Concurrently, intravenous methylprednisolone was transitioned to a 15-day oral prednisone taper. The patient was discharged in a stable condition on Day 21 for outpatient surveillance of CAAs and LTBI.

On Day 43, the patient was readmitted for anticoagulation conversion from subcutaneous enoxaparin to oral warfarin. Coronary computed tomography angiography (CCTA) confirmed persistent bilateral giant CAAs with early regression signs (**Figures 3A, B**; **Supplementary Videos 1**, **2**). To overcome rifampicin-induced warfarin resistance, we implemented an individualized pharmacokinetic-guided “prolonged bridging and meticulous titration” strategy. Warfarin was incrementally adjusted under therapeutic enoxaparin coverage with intensive INR monitoring (target INR: 1.5–2.5). A stable therapeutic INR was achieved within 2 weeks at a high maintenance dose of 3.125 mg/day, enabling a safe transition to warfarin monotherapy without thrombotic or hemorrhagic complications (**Figure 1B**). The patient was discharged on Day 58 on a final chronic management regimen consisting of warfarin combined with aspirin for dual antithrombotic therapy, and completion of a 3-month LTBI prophylactic course.

## Follow-up and outcome

**Figure 4:**
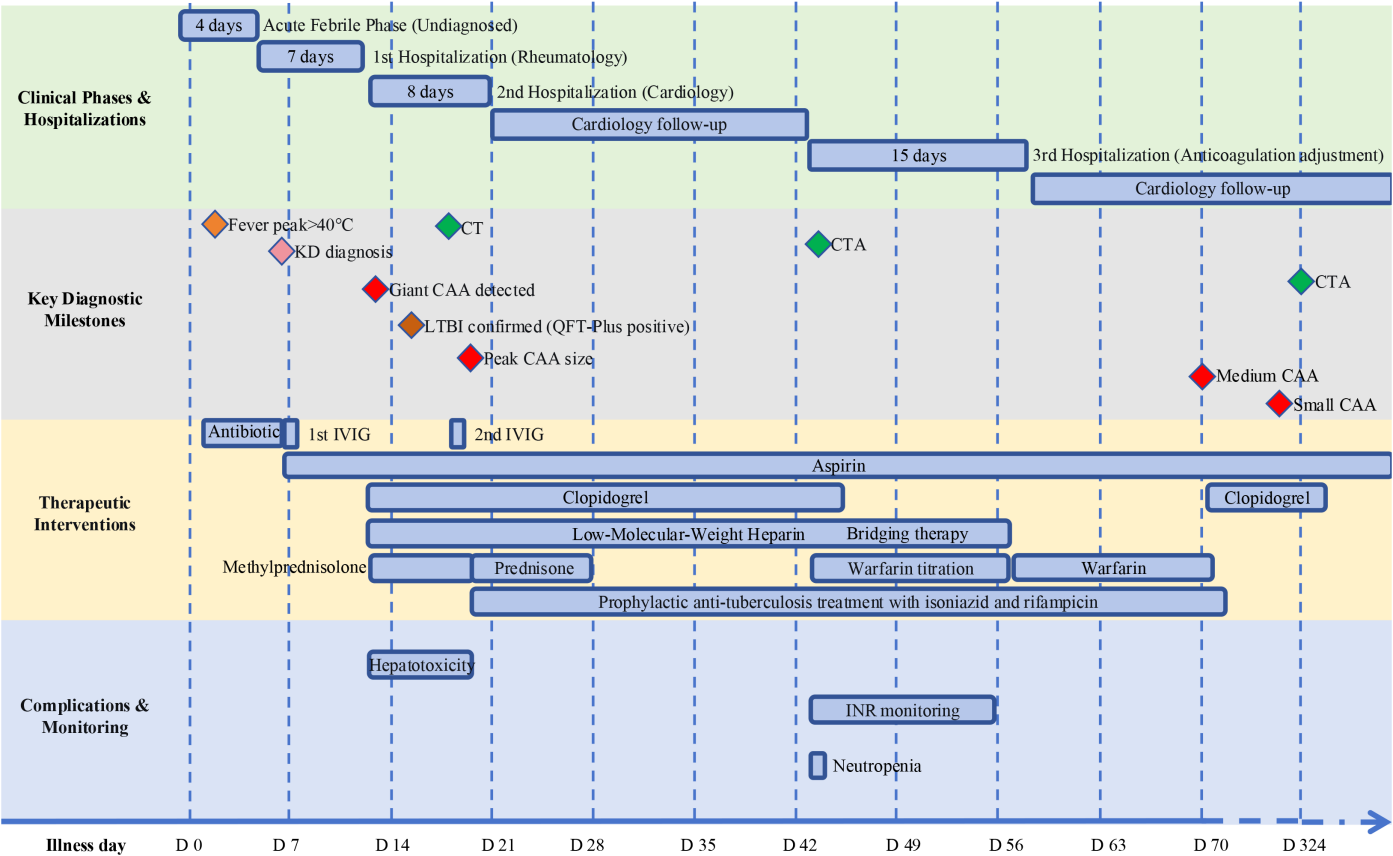
Timeline of clinical course, diagnostic milestones, therapeutic interventions, and complications.

After a 9-month follow-up, the patient achieved an excellent prognosis. Bilateral giant CAAs progressively regressed (**Figures 2A, B**); the Day 324 CCTA confirmed normalized luminal diameters without stenosis or aneurysms, despite slightly irregular contours (**Figure 3**; **Supplementary Video 3**). No severe adverse events (e.g., thrombosis, hemorrhage, LTBI reactivation) occurred. The overarching clinical timeline (**Figure 4**), serial laboratory/echocardiographic data, and the MDT decision-making process are detailed in **Supplementary Tables S1**-**S3**.

## Discussion

We present an extremely rare pediatric case of refractory KD complicated by giant CAAs and LTBI. Despite standard IVIG, rapid CAA progression necessitated intensified immunomodulation; however, concurrent LTBI strictly contraindicated guideline-recommended TNF-α inhibitors (1). Through an individualized, MDT-driven sequential regimen, the patient achieved an excellent prognosis. Herein, we analyze the immunopathological rationale and precision anticoagulation strategies underpinning this success, providing a practical framework for complex KD with severe CAAs and biologic contraindications.

Consistent with refractory KD immunopathogenesis, this patient exhibited synchronized hyperinflammation and hypercoagulability. Longitudinally, a disproportionate IL-6 surge (peaking at 868.83 pg/mL, Day 6) eclipsed adaptive cytokines (IL-2, IL-4, IFN-γ) (**Supplementary Table 1**), driving subsequent extreme thrombocytosis (821 × 10^9^/L, Day 13) and hepatic hs-CRP synthesis (**Figure 1A**). The precise 7-day kinetic lag between these peaks perfectly mirrors the biological timeframe for IL-6-driven megakaryopoiesis. This clinical triad strongly implicates a monocyte-driven state, corroborating recent single-cell RNA sequencing (scRNA-seq) findings that identify monocytes as the primary acute KD IL-6 source (11). Mechanistically, activated monocytes interact with hyperreactive platelets via adhesion molecules (e.g., P-selectin) to form platelet-monocyte aggregates (PMAs) (12), creating a synergistic positive feedback loop amplifying both inflammation and coagulation. Although direct PMA markers were unmeasured, the patient’s classic sequential cascade serves as a robust clinical surrogate. This “immune-coagulation crosstalk” coincided exactly with rapid CAA expansion. Ultimately, while adaptive immune dysregulation exists (11), this trajectory underscores the monocyte-platelet axis as the pivotal driver of early coronary vasculopathy.

Current recommendations for refractory KD suggest intensified immunomodulation with additional IVIG or combination therapy with GC or infliximab on an IVIG backbone (2). Though clinically potent (13), infliximab (a TNF-α inhibitor) is contraindicated in LTBI-positive patients, a rarely reported clinical dilemma in the literature. Yokoyama et al. highlighted this peril when LTBI was diagnosed post-infliximab initiation, exposing the child to severe tuberculosis reactivation risks, even though reactivation ultimately did not occur in this child (14). Conversely, our preemptive screening eliminated this hazard. Biologically, the rationale for this caution lies in the dual role of TNF-α: while monocyte-derived TNF-α drives the KD inflammatory cascade (11), it remains indispensable for macrophage recruitment and tuberculous granuloma integrity (15, 16). For LTBI-positive individuals like our patient, pharmacological TNF-α blockade disintegrates these granulomas, triggering active tuberculosis. This mechanistic hazard is substantiated by robust clinical and preclinical evidence, which confirms a markedly elevated risk of tuberculosis reactivation following TNF-α inhibition—a risk that can be significantly mitigated through standardized LTBI prophylaxis (17, 18). Collectively, these data underscore that systematic screening is not merely a formality but a mandatory safety pivot that dictates the selection of salvage immunomodulation.

Although systemic GCs pose a dose- and duration-dependent LTBI reactivation risk (19–22), we prioritized an IVIG-methylprednisolone regimen over infliximab due to fundamental mechanistic and pharmacokinetic advantages. Unlike infliximab, which physically clears TNF-α and induces cellular apoptosis, thereby risking rapid and irreversible granuloma disintegration (23), GCs functionally downregulate inflammation. Crucially, in contrast to the prolonged clearance of infliximab (weeks) (25), the short half-life of methylprednisolone (hours) (24) affords a highly controllable immunosuppressive window. This facilitated a strategic exit, allowing us to initiate prophylactic rifampin during the transition to tapering oral prednisone. As a CYP3A4 inducer, rifampin actively accelerates GC clearance, rapidly narrowing the high-risk window. Furthermore, infliximab mandates concurrent anti-TB prophylaxis, risking severe hepatotoxicity alongside high-dose aspirin. Should hepatotoxicity necessitate halting anti-TB drugs, residual infliximab would leave the patient defenseless against disseminated tuberculosis. In contrast, the rapid clearance of GCs offers a safe, immediate, and controllable exit strategy.

Following this IVIG-GC regimen, the patient’s inflammatory markers rapidly declined (**Figure 1A**) and CAA expansion halted (**Figure 2A**). Mechanistically, this aligns with recent scRNA-seq data (23) demonstrating that adding methylprednisolone to IVIG synergistically enhances the inhibition of monocyte-driven pathways (e.g., S100A-TLR signaling, NLRP3 inflammasome) and uniquely downregulates proinflammatory factors (e.g., CXCL5, IL-17C) resistant to IVIG monotherapy, providing a direct molecular basis for the patient’s rapid clinical stabilization. However, because repeated IVIG or IVIG-GC therapy elevates intra-CAA thrombotic risk (24, 25), establishing a precision anticoagulation strategy became our next clinical imperative.

Giant CAAs confer exceptional thrombotic risk, driven by immune–coagulation crosstalk (12) and evidenced by the patient’s acute thrombocytosis and hyperfibrinogenemia. While international protocols typically recommend an initial dose of 0.1 mg/kg/day with a target INR of 2.0–3.0 (26, 27), the Chinese consensus suggests a more cautious starting range of 0.05–0.12 mg/kg/day and a lower INR target of 1.5–2.5 (28), which likely reflects ethnic sensitivity in anticoagulant response. In strict accordance with this standard, warfarin was initiated for our patient at 1.25 mg/day (~0.078 mg/kg/day). However, rifampicin prophylaxis acts as a pharmacological double-edged sword. While its CYP450 induction strategically accelerated GC clearance, it simultaneously necessitated massive warfarin dose escalations (8, 9). To overcome this, we executed a rapid, percentage-based titration under intensive INR monitoring. Referencing CHEST principles (27), we aggressively escalated warfarin by 20–35% increments. Achieving a stable therapeutic INR ultimately required a 2.5-fold increase from the initial dose (to 3.125 mg/day, or ~0.195 mg/kg/day), a trajectory that quantifies the potent hepatic enzyme induction mediated by rifampicin.

To avert dangerous anticoagulant gaps, we strictly relied on a pharmacology-informed “prolonged LMWH bridge” strategy (10, 29). Therapeutic LMWH was maintained continuously throughout this titration window, strategically discontinued only after the INR safely entered the 1.5–2.5 range and stabilized on the 3.125 mg dose. During the maintenance phase, our protocol prioritized temporarily re-initiating LMWH for unpredictable INR dips rather than drastically altering warfarin, ensuring an uninterrupted antithrombotic shield. Ultimately, current KD guidelines (1) lack recommendations for LTBI-positive patients or concurrent rifampicin anticoagulation. Our case fills this gap by establishing a standardized workflow (**Figure 4**; **Supplementary Table 3**) encompassing pre-immunotherapy screening, alternative immunomodulation, timed prophylaxis, and pharmacology-guided anticoagulation, thereby providing practical evidence for future updates.

Prognostically, the apparent “resolution” of aneurysms on CCTA requires rigorous qualification. The 9-month luminal normalization likely represents “pseudonormalization” driven by intimal proliferation and chronic remodeling, rather than true anatomical restitution (30, 31). Furthermore, CCTA’s limited spatial resolution (~0.5 mm) may obscure subtle wall irregularities or nascent calcification. Because structural recovery does not equate to normalized endothelial function, these patients harbor profound long-term cardiovascular vulnerabilities. Long-term data starkly illustrate this threat: the 30-year overall survival is only 90%, while the cardiac event-free rate plummets to a dismal 36% (32). Therefore, lifelong cardiovascular surveillance remains mandatory to mitigate the persistent threats of premature atherosclerosis and late-phase complications (2).

The successful management of these profound clinical challenges critically depended on the synergistic collaboration of a structured MDT, comprising specialists from pediatric cardiology, rheumatology and immunology, gastroenterology, infectious diseases, and clinical pharmacy (**Supplementary Table 3**). This proactive, integrated model expertly navigated the delicate interplay of aggressive immunomodulation, intensive antithrombotic, and prophylactic anti-infective therapies. Consequently, the MDT ensured rigorous clinical safety—averting coronary thrombosis, severe hemorrhagic events, LTBI reactivation, and hepatotoxicity—while achieving exceptional therapeutic efficacy, evidenced by remarkable vascular remodeling, the regression of bilateral giant CAAs, and the preservation of normal cardiac function. Ultimately, this case underscores that for patients with refractory KD and complex pharmacological comorbidities, establishing a structured, data-driven multidisciplinary care pathway is indispensable for optimizing long-term clinical outcomes.

## Conclusion

This case illustrates how an MDT-led, pathophysiology-driven strategy can successfully navigate the “triple challenge” of refractory KD, giant CAAs, and LTBI. By executing an alternative IVIG-GC regimen, strategically timing tuberculosis prophylaxis, and implementing pharmacology-guided precision anticoagulation, our approach elegantly balanced the competing demands of profound inflammation, exceptional thrombotic risk, and infection safety. Ultimately, this integrated framework provides a practical clinical paradigm for managing intricate immune-coagulation-infection crosstalk, offering foundational evidence to inform future guidelines for high-risk KD.

## Limitations

Despite the favorable outcome, several inherent limitations warrant consideration. First, as a single-center report, the generalizability of our MDT-led protocol requires multi-center validation to account for inter-individual variability in disease progression and drug responses. Second, the profound rifampicin-warfarin interaction was inferred pharmacodynamically via serial INR monitoring, lacking direct pharmacokinetic quantification (e.g., serum warfarin levels or CYP450 enzyme activity). Third, while the patient’s clinical trajectory (IL-6 and platelet surges) strongly supported the monocyte-platelet axis hypothesis, we lacked individualized, direct immune profiling. Future validation necessitates techniques like flow cytometry or patient-specific scRNA-seq to definitively quantify transient pathogenic subsets and interaction markers, such as CD16+ monocytes, P-selectin, and PMAs. Ultimately, while this case establishes a practical paradigm for complex KD, prospective studies integrating individualized pharmacokinetic mapping and multi-omic profiling are essential to refine and universally validate these interventions.

## Patient perspective

When our 3.5-year-old son was diagnosed with refractory KD and giant CAAs, complicated by a concurrent LTBI, we were extremely anxious. The treatment process was complex, requiring frequent blood draws and daily subcutaneous injections, which was heartbreaking to witness. However, the MDT explained the necessity of every step with great patience and transparency. Recognizing the life-threatening nature of his condition, we decided to trust the team completely and cooperate fully with the rigorous treatment plan. We strictly adhered to the complex medication schedules, particularly the precise dosing of his blood thinners and tuberculosis prophylaxis. We also meticulously attended all follow-up appointments. Seeing his CAAs regress significantly after 9 months brings us immense relief. We fully understand the necessity of lifelong cardiac monitoring and remain committed to strict medication compliance to protect his long-term health.

## Data Availability

The original contributions presented in the study are included in the article/**Supplementary Material**. Further inquiries can be directed to the corresponding authors.
